# Tunable Carrier Type of a Semiconducting 2D Metal–Organic Framework Cu_3_(HHTP)_2_

**DOI:** 10.1021/acsami.2c00089

**Published:** 2022-03-01

**Authors:** Maria de Lourdes Gonzalez-Juarez, Carlos Morales, Jan Ingo Flege, Eduardo Flores, Marisol Martin-Gonzalez, Iris Nandhakumar, Darren Bradshaw

**Affiliations:** † School of Chemistry, University of Southampton, Southampton SO17 1BJ, U.K.; ‡ Applied Physics and Semiconductor Spectroscopy, Brandenburg University of Technology Cottbus−Senftenberg, Konrad-Zuse-Strasse 1, D-03046 Cottbus, Germany; § Instituto de Micro y Nanotecnología (IMN-CNM-CSIC), C/ Isaac Newton 8, PTM, E-28760 Tres Cantos, Spain; ∥ Centro de Nanociencias y Nanotecnología (CNyN), Universidad Nacional Autónoma de México (UNAM), Ensenada, Baja California C.P. 22860, Mexico

**Keywords:** conducting, metal−organic frameworks, molecular doping, n-type, p-type

## Abstract

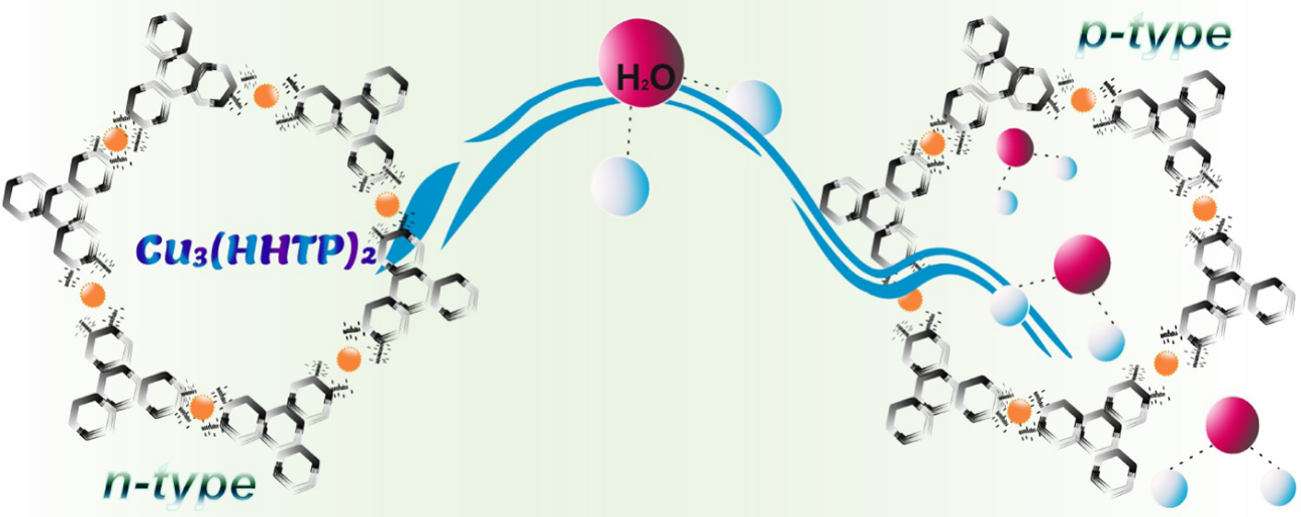

In this work, a switch from n-type to p-type conductivity in electrodeposited Cu_3_(2,3,6,7,10,11-hexahydroxytriphenylene)_2_ [Cu_3_(HHTP_2_)] has been observed, which is most likely due to oxygen molecular doping. The synthesis of electrically conductive 2D metal–organic frameworks (MOFs) has been achieved through the introduction of highly conjugated organic linkers coordinated to their constituent metal-ion centers. However, the porous structure and unsaturated metal sites in MOFs make them susceptible to ambient adsorbates, which can affect their charge transport properties. This phenomenon has been experimentally investigated by GIXRD, Hall effect and Seebeck measurements, and X-ray photoelectron spectroscopy.

## Introduction

Metal–organic frameworks (MOFs) are porous materials composed of inorganic and organic building blocks. These consist of metal ions coordinated to organic ligands, resulting in a wide range of 2D or 3D structures. The porosity and high surface area of these materials are promising properties that make them suitable for gas separation,^1^ catalysis,^2^ and chemiresistive gas sensor^3^ applications. The majority of the MOF structures behave as electrical insulators because of the lack of overlap between the metal and ligand frontier orbitals, leading to poor charge transport properties. For inherent MOF insulators, postsynthetic routes have been employed to enhance their electrical conductivity. Taking advantage of their porosity, the introduction of redox-active guest molecules into the MOF pores has been used as a method to generate pathways for charge transfer. For instance, the electrical conductivity of TCNQ@HKUST-1 (where the redox molecule TCNQ = tetracyanoquinodimethane) was enhanced by >7 orders of magnitude in this way.^4^ However, based on the variety of possible combinations of metal clusters and organic linkers available to date, it is possible to synthesize intrinsic electrically conductive MOFs. The effective charge transport in these MOFs arises from the π-electron delocalization originating from electroactive or highly conjugated aromatic ligands that overlap with the metal d-orbitals. Furthermore, π-stacking in close proximity to electroactive ligands within the framework also provides a path for free charge carriers.^5^ Examples of these MOFs are those in which their architectures resemble that of graphene. These frameworks comprise trigonal hexamino-, hexahydroxy-, and hexathio-substituted benzene or triphenylene linkers coordinated to square planar metal ions (e.g., Ni^2+^, Co^2+^, Cu^2+^), leading to an extended two-dimensional porous crystalline structure. Comprehensive reviews regarding the charge transport regimes and design strategies in conductive MOFs can be found in the literature.^6−9^ However, the important features in semiconductor materials, such as the majority charge carrier type (i.e., p- or n-type, for holes and electrons, respectively), carrier density, and charge mobility, have been barely investigated experimentally in conductive MOFs. The determination of these parameters is important to boost the potential application of conductive MOFs in electronic components (e.g., transistors, diodes, etc.) or devices for energy harvesting, such as photovoltaics and thermoelectrics. In practice, this data is extracted via either field-effect transistors (FETs) or Hall effect measurements. Furthermore, Seebeck measurements can be carried out to determine the majority carrier type. The sign of the Hall voltage indicates the majority of charge carriers in the sample, being positive or negative for holes and electrons, respectively. Finally, the Seebeck effect involves the transport of charge carriers by applying a temperature difference (Δ*T*) across the material. Charge carriers travel from the hot to the cold side generating a voltage differential (Δ*V*), also known as the Seebeck voltage. The Seebeck coefficient *S* is then calculated from the ratio of the generated thermopower and temperature difference (*S* = −Δ*V*/Δ*T*). Depending on the dominant charge carrier type in the material, the Seebeck coefficient can be positive (holes, p-type) or negative (electrons, n-type), employing the Telkes’ criterion for the voltage and the sign of the Seebeck coefficient.^10^

**Figure 1:**
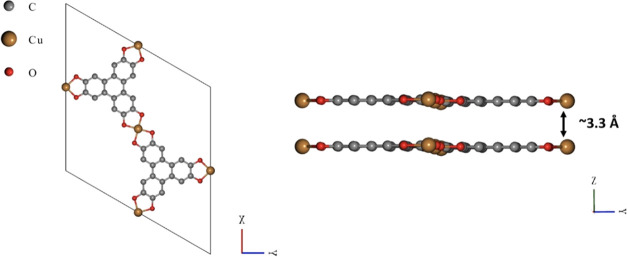
Connectivity and packing of the Cu_3_(HHTP)_2_ framework.

The large surface area and porosity of conductive MOFs may result in interactions or weak bonding with molecules from the surrounding environment (e.g., H_2_O, O_2_) and the framework, leading to variations in their electrical response.^5^ Therefore, when evaluating the electrical properties of MOFs, it is important to provide details about the conditions (e.g., atmosphere/vacuum, illumination/dark, and temperature) under which the measurements were carried out for better comparative studies. Cu_3_(2,3,6,7,10,11-hexahydroxytriphenylene)_2_ [Cu_3_(HHTP)_2_] is a semiconductive framework, which has been widely investigated for chemiresistive sensors^11−14^ and lately for Li- and Zn-ion battery applications.^15,16^ The molecular building units of Cu_3_(HHTP)_2_ comprise Cu ions coordinated with a 2,3,6,7,10,11-hexahydroxytriphenylene (HHTP) redox-active ligand, leading to extended 2D sheets stacked along the crystallographic *c* direction (Figure 1). The pore size and interlayer spacing between its AA packed sheets are ∼18 and 3.3 Å, respectively.^17^

The motivation for the current work is based on our previous study, which reported the synthesis of Cu_3_(HHTP)_2_ by electrochemical routes and determination of its thermoelectric properties.^18^ To perform thermoelectric measurements, a wet chemical method was employed to transfer the electrochemically synthesized MOF films to remove the electrical contribution of the conducting substrate onto which the films were initially electrodeposited. A suspension of poly(methyl methacrylate) (PMMA) dissolved in chlorobenzene was used as a transfer agent. The resultant negative Seebeck coefficients measured at room temperature in bulk and thin films suggested that the majority of the charge carriers in the material were electrons; thus, Cu_3_(HHTP)_2_ behaves as an n-type semiconductor. An interesting finding with reproducible results was observed when the drop-casted PMMA suspension on the MOF film was dried at higher temperatures; the sign of the Seebeck coefficient switched from negative to positive, resulting in a value of +269.5 μV K^–1^. This experimental ambipolar behavior has also been reported for Ni_3_(2,3,6,7,10,11-hexaiminotriphenylene)_2_ (Ni_3_[HITP]_2_), a structurally analogous framework of Cu_3_(HHTP)_2_, where a p-type and n-type behavior was experimentally obtained in FET^19^ and Seebeck measurements,^20^ respectively. The concept of doping has been widely covered in numerous publications on the physics of inorganic semiconductors. Doping differs when the same concept is applied to organic semiconductor materials. For instance, in inorganic semiconductors, doping refers to the substitution of atoms in a covalently bound lattice. This is widely observed in practice with the p- and n-type doping of silicon, which is generally conducted by the introduction of boron or phosphorous impurities into the Si lattice, respectively. On the other hand, the phenomenon of charge transfer between molecular components in an organic-material-based film that consequently leads to different electronic interactions is denoted as intermolecular doping.^21^

In the present work, we systematically investigate the molecular doping of Cu_3_(HHTP)_2_ derived from the wet chemical method employed for the film transfer, causing a switch in the carrier type (i.e., from n- to p-type conduction). The methodology for the hydrothermal and electrochemical synthesis of Cu_3_(HHTP)_2_ is reported in our previous work.^21^ Charge transport properties such as carrier type, electron mobility, and charge mobility in bulk pressed-powder and thin-film configurations were investigated by Seebeck and Hall effect measurements under ambient conditions. XPS measurements revealed important features regarding the Cu/O ratio in the transferred electrodeposited MOF thin-film samples.

## Results and Discussion

To the best of our knowledge, the carrier type in Cu_3_(HHTP)_2_ has only been studied by ultraviolet photoelectron spectroscopy (UPV)^11^ and FET measurements,^22^ indicating a p-type semiconducting character. It is noteworthy to highlight that these measurements were conducted on 10 nm thick films under ambient conditions. We believe this p-type behavior may be attributable to the physisorption of O_2_ or H_2_O molecules from the air into the framework, which could impact its electrical response. Furthermore, according to X-ray photoelectron spectroscopy (XPS) measurements, an excess of O_2_ is reported in Cu_3_(HHTP)_2_-based FET devices.^22^ Since molecular oxygen has been demonstrated to act as an electron acceptor, its role as a p-type dopant has also been reported for 1D and 2D architectures in FET-based devices such as graphene,^23^ carbon nanotubes,^24^ MoTe_2_,^25^ WSe_2_, and MoSe_2_.^26^ In addition, the p-doping effect observed particularly in FET devices comprising films that are several nanometers in thickness cannot result exclusively from the interaction of the material with the surrounding environment, but because of the chemical nature of the substrate (e.g., silanol or hydroxyl groups attached to the SiO_2_/Si surface).^27^ For instance, the presence of ambient adsorbates on an SiO_2_ substrate surface in FET devices has been shown to induce a reduction or suppression in the n-type conduction. This phenomenon might cause discrepancies when reporting the intrinsic properties of the material. A similar case is that of Ni_3_(HITP)_2_, where FET devices made of 105 nm thick MOF films exhibited a p-type character, but a negative Seebeck coefficient was obtained from thermoelectric measurements conducted on an Ni_3_(HTIP)_2_ pellet, suggesting n-type conduction.^20^ This ambipolar behavior was experimentally observed for Cu_3_(HHTP)_2_, in which Seebeck measurements conducted on MOF pellets (Ø 13 × 0.8 mm^2^) and 5 μm thick films showed that electrons act as majority carriers in the material^18^ leading to n-type conduction, contrary to what is reported in the literature.^11,22^ However, recently, Ninawe et al. demonstrated the stability and conversion from n-type to p-type conduction of bulk Cu_3_(HHTP)_2_ upon the chemical integration of reduced graphene oxide (rGO).^28^

In this paper, we discuss the change in the semiconducting character of electrochemically deposited MOF films before and after being transferred with a PMMA support for their further characterization.

Briefly, the electrochemical synthesis of Cu_3_(HHTP)_2_ was conducted on Au/SiO_2_ substrates. A copper layer (area 1 cm^2^) was electrodeposited onto Au/SiO_2_ for its subsequent anodic dissolution to provide Cu ions to be coordinated with the organic linker HHTP in solution (SI-Methods). As every electrochemical experiment requires a conductive substrate as a working electrode, to perform thermoelectric measurements and avoid the electrical contribution from the substrate, the MOF film was transferred using a PMMA suspension. PMMA dissolved in chlorobenzene was drop casted onto MOF@Au/SiO_2_ and subjected to a drying process at 40 or 70 °C overnight. This transfer method results in the MOF film adhering to the PMMA layer, which can be easily detached from the Au/SiO_2_ substrate by simply peeling it away, leaving the Cu_3_(HHTP)_2_ thin film supported on PMMA. Before investigating the thermoelectric properties or further characterization of the transferred Cu_3_(HHTP)_2_ films, it is important to determine whether the transferred MOF films are electrically conductive. Because of the porosity of the electrodeposited film, PMMA may diffuse through the film, leading to a PMMA-embedded MOF film. The electrical test can be conducted by simply using a multimeter and assuring that there is an electrical response between the two probes.

**Figure 2:**
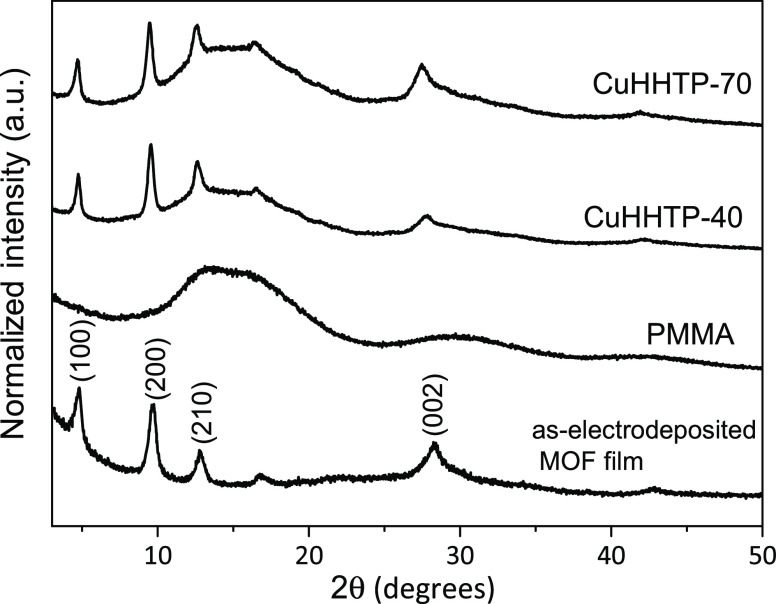
GIXRD patterns of pristine electrodeposited Cu_3_(HHTP)_2_ on SiO_2_, neat PMMA support, and PMMA-transferred Cu_3_(HHTP)_2_ thin films dried at 40 °C (CuHHTP-40) and 70 °C (CuHHTP-70).

The successful electrodeposition and crystalline phase of Cu_3_(HHTP)_2_ was confirmed by grazing incidence X-ray diffraction (GIXRD), as shown in Figure 2. The diffraction peaks located at 4.79, 9.56, 12.59, and 27.86° correspond to the (100), (200), (210), and (002) crystal domains, respectively. This data is in agreement with the diffraction pattern of bulk Cu_3_(HHTP)_2_.^29^ In addition, GIXRD measurements were conducted before and after the transfer process with PMMA. The diffraction patterns labeled as CuHHTP-40 and CuHHTP-70 correspond to the transferred Cu_3_(HHTP)_2_ films, with the drop-casted PMMA layer dried at 40 and 70 °C, respectively. Well-defined diffraction peaks corresponding to the MOF phase in the transferred electrodeposited films suggest that the MOF structure is not compromised during the transfer process. The broad peak located in the 2θ range of 10–20°, in both transferred samples, is due to the contribution of the amorphous phase of the polymer acting as the MOF film support.

**Figure 3:**
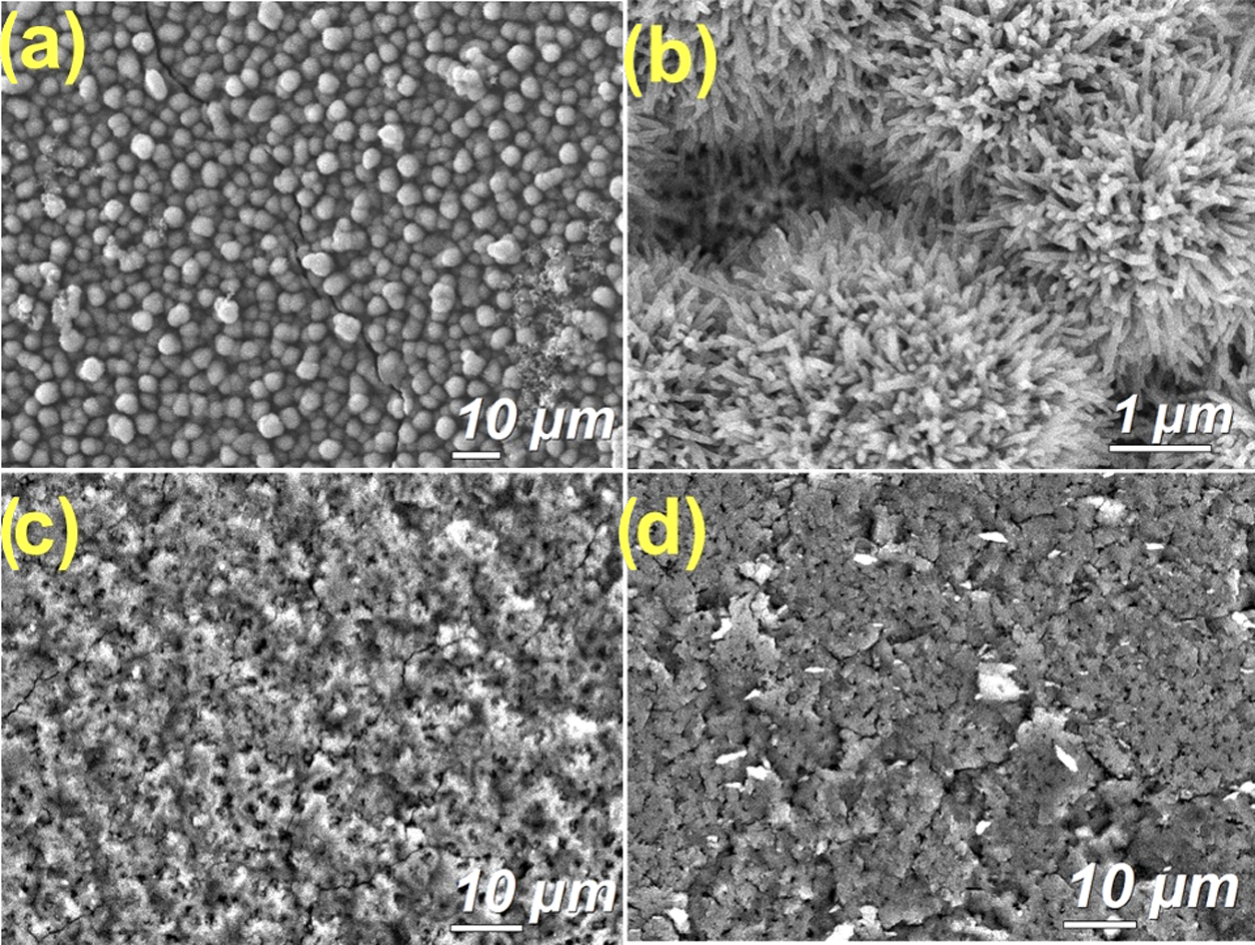
SEM images of as-electrodeposited Cu_3_(HHTP)_2_ (a, b) and transferred MOF films dried at 40 °C (c) and 70 °C (d).

Microstructure, cross-sectional, and elemental analysis of the electrodeposited Cu_3_(HHTP)_2_ thin films before and after the transfer process was carried out by scanning electron microscopy (SEM) (Figure 3). In contrast to the hydrothermal synthesis of Cu_3_(HHTP)_2_, the morphology resulting from the electrochemical deposition of this framework consists of stacked spherical particles with diameters of ∼6 μm (Figure 3a). Higher-magnification imaging allowed the identification of aggregated nanorods with average widths of 86 nm. These were identified to be part of the structural composition of these spherical MOF particles (Figure 3b). From SEM cross-sectional measurements, the thickness of the electrodeposited Cu_3_(HHTP)_2_ was estimated to be ∼5 μm (Figure S1). The chemical composition of the Cu_3_(HHTP)_2_ film was investigated by energy-dispersive X-ray spectroscopy (EDS). The elemental analysis shows the presence of Cu, O, and C (Figure S2), in which the atomic ratio is in good agreement with the composition of the framework Cu_3_C_36_H_18_O_12_. The SEM images of the PMMA-transferred MOF films dried at 40 and 70 °C are shown in Figure 3c,d. For better image acquisition, samples were coated with a 5 nm gold layer to reduce the effect of beam charging in some areas, likely to be caused by PMMA residues on the surface of the sample. The CuHHTP-40 film sample shows a flattened and compact surface with clear interparticle connectivity (Figure 3c). Some difficulties were encountered during cross-sectional imaging due to the insulating nature of the PMMA residues due to beam charging of the sample, especially at higher magnifications. For this reason, the interface of the MOF/PMMA layer was hard to differentiate. EDS analysis reveals the presence of chlorine, which may be attributed to the chlorobenzene employed to dissolve the PMMA during film transfer. On the other hand, a rougher surface is observed for the CuHHTP-70 sample (Figure 3d). This is presumably due to the different rates of evaporation for the chlorobenzene during the drying process (and/or shrinking of the drop-casted PMMA layer occurring at higher temperatures^30^), leading to the observed differences in film corrugation. Despite the patchy appearance of the MOF film, successful electrical measurements were conducted on both transferred samples.

## Charge Transport Properties

**Figure 4:**
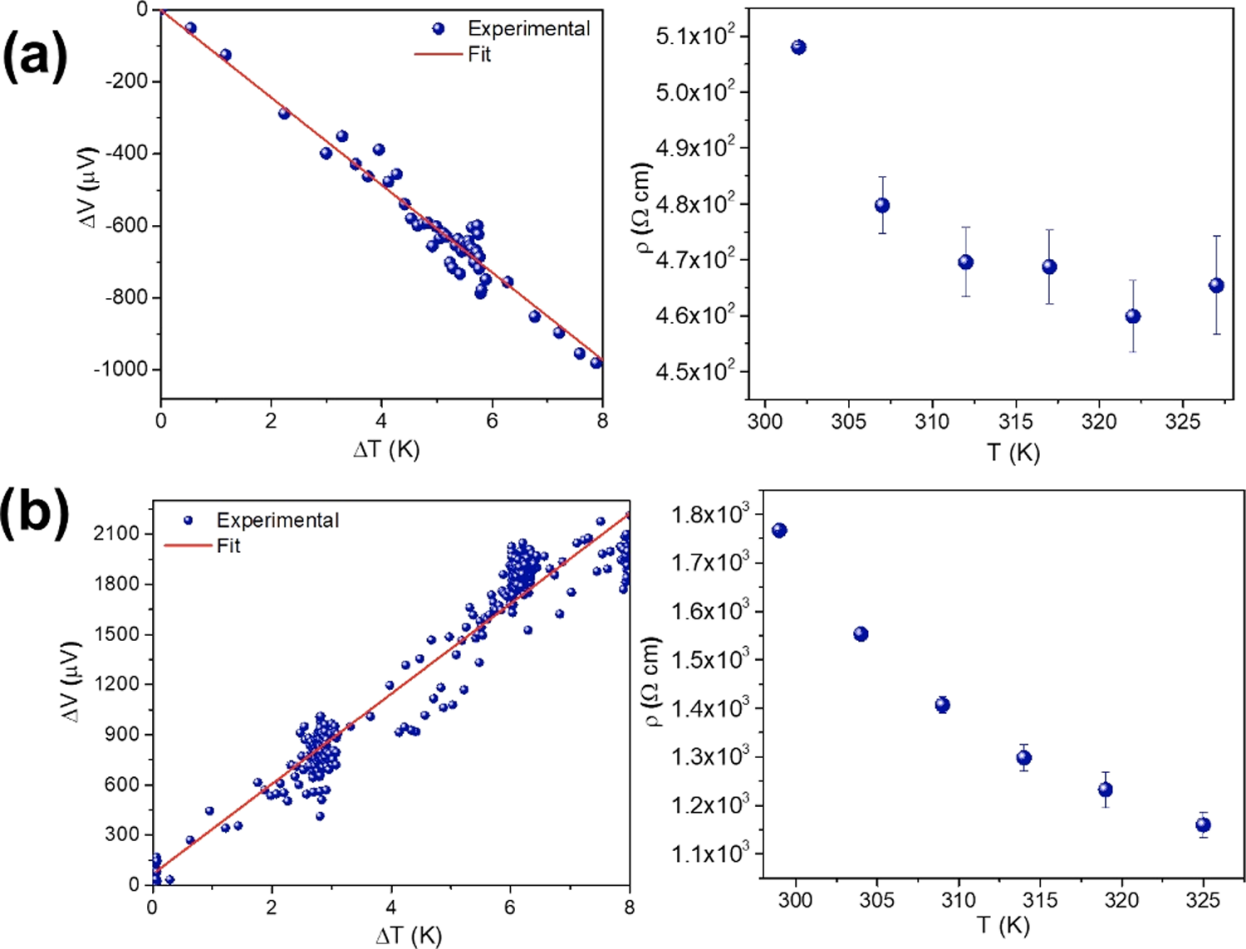
Thermopower and temperature-dependent electrical conductivity data for CuHHTP-40 (a) and CuHHTP-70 (b).

The electrical conductivity and charge transport properties of CuHHTP-40 and CuHHTP-70 transferred films were evaluated by four-point probe and Hall effect measurements at room temperature, respectively. The electrical contacts between the MOF film and the probes of the instrument were qualitatively assessed by performing current–voltage (*I*–*V*) measurements (Figure S3). Despite some PMMA residues being observed on the MOF films derived from the transfer process, the linear *I*–*V* behavior suggests a good Ohmic contact that permits electrical measurements to be conducted. The semiconducting nature of Cu_3_(HHTP)_2_ was confirmed by temperature-dependent electrical conductivity measurements (Figure 4), where a decrease in resistivity upon increasing the temperature was observed. From four-point probe electrical measurements, conductivities of 2.28 × 10^–3^ and 4.86 × 10^–4^ S cm^–1^ were recorded for CuHHTP-40 and CuHHTP-70, respectively. The 5-fold difference in conductivity between the two MOF-transferred films is presumably due to the microstructure and quality of the surface of the MOF films.^31^ Microstructure,^32^ surface roughness,^33^ and coating crystallinity degree^34^ are physical features of thin films that have a significant impact on their electrical properties. CuHHTP-70 exhibits a more disrupted surface compared to CuHHTP-40 as indicated by SEM. The lower conductivity observed for CuHHTP-70 therefore could be attributed to the resistivity contribution derived from the particle interfaces acting as electron scattering sites. This is supported by charge transport values determined by Hall effect measurements. The charge carrier concentrations of CuHHTP-40 and CuHHTP-70 films are −6.41 × 10^16^ and +2.47 × 10^14^ cm^3^, respectively. The electrical conductivity σ is proportional to the product of the carrier concentration and mobility, as shown by the expression σ = *ne*μ, where σ, *n*, *e*, and μ correspond to electrical conductivity, carrier density, elementary charge (i.e., holes/electrons), and carrier mobility, respectively. The experimentally measured *n* values for both transferred MOF films are in good agreement with the conductivity values, where σ_CuHHTP-40_ > σ_CuHHTP-70_. The low charge carrier concentration observed in CuHHTP-70 could be attributed to the higher thermal treatment (i.e., 70 °C), where positive charges from broken chemical bonds in PMMA were formed causing a decrease in the electron mobility as it has been demonstrated in Al/PMMA/p-Si structures.^35^ Furthermore, a higher temperature could lead to the diffusion of PMMA through the CuHHTP-70 film, forming a thin layer of PMMA on the MOF thin-film surface. For instance, electron microscopy images of the CuHHTP-70 film surface (Figure 3d) showed brighter zones that can be attributed to the electron charging of PMMA residues. This PMMA layer could passivate the surface of the MOF film and act as an electron trap, thus hindering electronic charge transport.

Seebeck measurements were carried out as a complementary approach to investigate the carrier type of the PMMA-transferred Cu_3_(HHTP)_2_ films. Based on the measurement from five independent films at each drying temperature, CuHHTP-40 exhibited an average negative Seebeck coefficient (α) of −117.0 ± 13.4 μV K^–1^ (Figure 4a), suggesting electrons are the majority charge carriers in the material. On the other hand, the positive slope observed in CuHHTP-70 (Figure 4b) indicated that this sample behaves as a p-type semiconductor with an average Seebeck coefficient of +269.5 ± 21.6 μV K^–1^.

The absolute high Seebeck coefficient recorded for CuHHTP-70 can be explained, prima facie, by its carrier concentration. The lower the charge carrier density, the higher the Seebeck coefficient, according to Mott’s relation , where *T* is the temperature, *n*(*E*) is the carrier density at energy *E*, μ(*E*) is the mobility at energy *E*, *E*_F_ is the Fermi level, *k*_B_ is the Boltzmann constant, and *q* is the electronic charge. Therefore, *n*_CuMOF40_ > *n*_CuMOF70_ according to carrier concentration values stated above correlates to α_CuHHTP-40_ < α_CuHHTP-70_.

**Figure 5:**
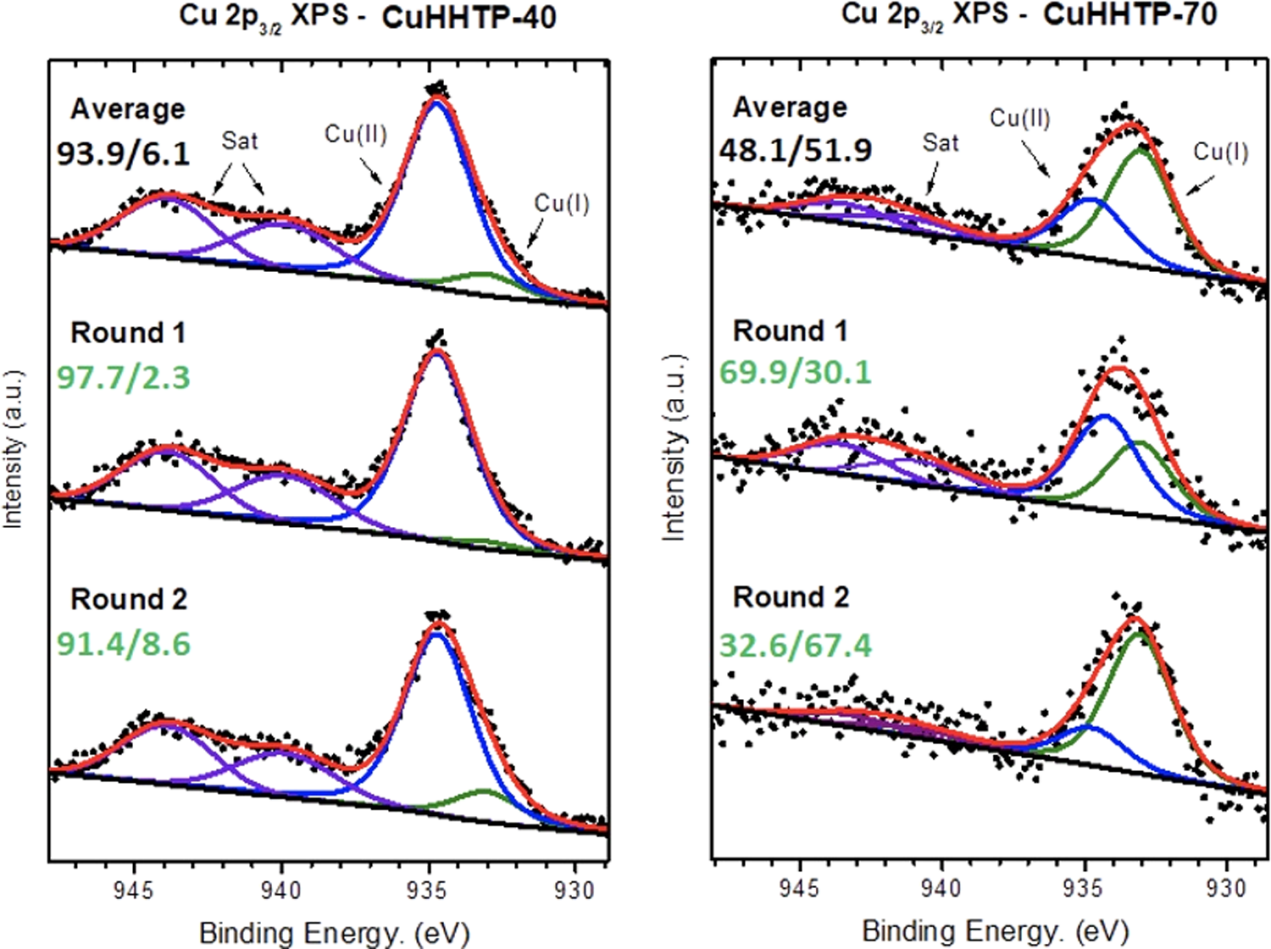
XPS spectra of Cu 2p_3/2_ of the transferred MOF films CuHHTP-40 and CuHHTP-70. Numerals indicate the Cu^II^/Cu^I^ ratios at the beginning (Round 1) and end (Round 2) of the measurement. The top spectra show the average between both sets of measurements and the average Cu^II^/Cu^I^ ratio obtained.

X-ray photoelectron spectroscopy (XPS) was employed to investigate the chemical composition and valence state of the Cu species in the CuHHTP-40 and CuHHTP-70 samples. Atomic concentrations (i.e., Cu, C, O) were estimated from XPS survey spectra, suggesting that both CuHHTP-40 and CuHHTP-70 are closer to the theoretical value Cu 5.9% C 70.6% O 23.5% for Cu_3_(HHTP)_2_ (Figure S4). On the other hand, the analysis conducted on the Cu 2p region spectra revealed important variations in the Cu(II)/Cu(I) ratio for CuHHTP-40 and CuHHTP-70, showing that the latter exhibited a significant reduction in Cu(II) species (Figure 5). In principle, correlating the Cu(II)/Cu(I) ratio with electrical conductivity measurements, where σ_CuHHTP-40_ > σ_CuHHTP-70_, an unfilled Cu(II) d-valence shell provides a more delocalized electronic structure compared to d^10^ Cu(I).^36^ Thus, a reduction in Cu(II) species would result in a weaker orbital overlap with the linker moieties limiting the MOF charge transport properties. In addition, the O/Cu ratio is 4 times higher in CuHHTP-70 than that in CuHHTP-40 (Table S1). This finding could provide insights into why we observe n-type and p-type conduction in CuHHTP-40 and CuHHTP-70, respectively.

Interestingly, CuHHTP-70 exhibited less stability than CuHHTP-40 when exposed to the X-ray source for XPS data collection. A significant reduction in the ratio of Cu^II^/Cu^I^ as a function of the X-ray exposure time was observed for CuHHTP-70. Two subsequent sets of 50 scans were measured to monitor the X-ray beam damage, differentiating the MOF initial and final chemical states after X-ray exposure (Figure 5). For instance, the ratio of Cu^II^/Cu^I^ (%) in CuHHTP-40 significantly decreases from 97.7/2.3 to 91.4/8.6, with a total exposure time of 90 min in each set. The Cu^II^/Cu^I^ ratio in CuHHTP-70 varied from 69.9/30.1 to 32.6/67.4 over the same time interval, with Cu(I) becoming dominant.

PMMA has been widely used as a supporting polymer in wet transfer processes for thin films, particularly for 2D materials.^37−39^ However, in most instances, the complete removal of PMMA from the surface of these materials is still a challenge, having a direct impact on their physical properties. For instance, a decrease in the electron mobility of graphene monolayers due to PMMA residues has been reported by Suk et al.^39^ This phenomenon has been attributed to PMMA residues increasing the external scattering sites and altering the electron delocalization of the material, inducing a p-type doping effect.^39^ Furthermore, the insulating nature of PMMA arising from its wide HOMO–LUMO energy gap (5.6 eV) makes it a potential charge-blocking material.^40^ It is noteworthy to mention that PMMA is a neutral molecule that possesses neither electron-withdrawing groups nor electron-donating groups^41^ and should not be considered as a dopant agent. Nonetheless, in PMMA composites, the carbonyl group present in PMMA can act as an electron-accepting group leaving positively charged holes as charge carriers, as observed in organic semiconductors.^42^ On the other hand, p-type doping caused by the adsorption of molecular O_2_ from air/moisture, which acts as an electron acceptor, is likely to occur according to the mechanism described by Aguirre et al.^27^ They considered the molecular orbital energies of the organic semiconductor material in the study (e.g., carbon nanotubes) and the electrochemical potential of the oxygen/water redox couple. The electron transfer occurs when the highest occupied molecular orbital (HOMO) energy level in the organic semiconductor aligns with the electrochemical potential of the O_2_/H_2_O redox couple favoring p-type conduction. In this context, the calculated HOMO energy level of Cu_3_(HHTP)_2_ is −5.32 eV, according to ultraviolet photoemission spectroscopy (UPS) studies conducted under air condition.^29^ The electrochemical potential of the O_2_/H_2_O redox couple is −5.3 eV.^27^ Therefore, the possibility of hole doping induced by the presence of oxygen/water from the environment is feasible according to the mechanism described earlier. The water adsorption affinity of Cu_3_(HHTP)_2_ and its impact on the electrical properties of the framework have been investigated via molecular dynamics and band structure calculations.^43^ The adsorption of water molecules in Cu_3_(HHTP)_2_ has been predicted to occur initially through the formation of hydrogen bonds with HHTP ligands due to their constituent oxygen atoms. Subsequently, water molecules penetrate the framework and form coordinative bonds with open Cu^2+^ centers. The band diagrams and projected density of state (pDOS) calculations revealed that even the presence of only 1 water molecule between the MOF layers results in important changes in charge mobility since through-space π–π interactions along the *c* direction are reduced due to the increase of interlayer distance. Furthermore, water molecules within the MOF are likely to act as charge carrier traps, as has been suggested from the displayed curvatures in the conduction band of the modeled hydrated MOF structure.^43^ Therefore, the excess of O observed in CuHHTP-70 could be attributed to the presence of molecular water from the environment adsorbed within the MOF pores, as has been previously reported.^22,44^ In addition, a more hydrated MOF sample would exhibit a lower Cu(II)/Cu(I) ratio due to the preferential adsorption of water molecules through hydrogen-bonded interactions at Cu^2+^ sites,^45^ a fact that is in good agreement with our estimated Cu(II)/Cu(I) ratios.

Charge mobility values extracted from Hall Effect measurements were 0.364 and 0.298 cm^2^ V^–1^ s^–1^ for CuHHTP-40 and CuHHTP-70, respectively. The electronic mobility of the samples can be reduced either by the surface scattering effects derived from grain boundaries or by impurity scattering, such as PMMA residues presumably present in both transferred MOF film samples as observed by SEM characterization. However, these factors are predominantly present in the CuHHTP-70 film sample, as suggested by electrical and microstructure characterizations.

The effect of carrier-type switch of Cu_3_(HHTP)_2_ thin films was also investigated by varying the polymer and solvent used as transfer agents by employing polyacrylic acid (PAA) powder dissolved in methanol. The solution was left to stir at room temperature overnight to ensure a homogeneous mixture, and the methodology to transfer the electrodeposited Cu_3_(HHTP)_2_ films was the same as described for PMMA. Following drop casting of PAA/MeOH onto electrodeposited Cu_3_(HHTP)_2_ films, these were also dried at 40 and 70 °C (Figure S5). Electrical measurements on these films were highly reproducible and reveal a similar switching behavior, which suggests the change in carrier type observed is not restricted to the use of PMMA as a transfer agent alone, indicating the potential generalizability of our approach (cf. Table S2 and Figure S6 in the SI).

## Conclusions

In conclusion, we report a switch in the semiconducting character of a 2D semiconducting framework thin film, Cu_3_(HHTP)_2_, in which its conduction type (n-type) seemed to be affected by the excess of molecular oxygen within the framework, leading to p-type conduction as demonstrated by XPS and Seebeck measurements. In addition, the hole doping effect is theoretically addressed by the energy alignment of the water/oxygen redox couple and the HOMO level of Cu_3_(HHTP)_2_. Further investigations involving molecular dynamics and band structure calculations of this system are ongoing to confirm potential molecular doping. The crystal structure of the electrodeposited Cu_3_(HHTP)_2_ thin films after being transferred with PMMA is not compromised, as suggested by GIXRD characterization. The charge transport properties of the MOF film are affected due to a residual PMMA layer on the MOF film surface as observed in SEM. P-type Cu_3_(HHTP)_2_ was found to be less stable toward X-ray exposure, which resulted in reduction of more Cu^II^ to Cu^I^ species.

## Supporting Information Available

Experimental methods (materials); electrochemical deposition of Cu_3_(HHTP)_2_ onto Au/SiO_2_; Cu_3_(HHTP)_2_ thin films transferred with PMMA; characterization methods; SEM cross-section of Cu_3_(HHTP)_2_ onto Au-coated SiO_2_; EDS spectrum of the electrodeposited Cu_3_(HHTP)_2_ film; *I*–*V* curves of transferred Cu_3_(HHTP)_2_ thin films; XPS spectra of transferred CuHHTP-40 and CuHHTP-70 films; atomic concentrations in elemental region (Cu, C, O) from XPS survey spectra (Table S1); Cu_3_(HHTP)_2_ thin films transferred with PAA; photographs of Cu_3_(HHTP)_2_ film using PAA; thermopower data of electrodeposited Cu_3_(HHTP)_2_ films transferred with PAA; and charge transport data for electrodeposited Cu_3_(HHTP)_2_ films using PMMA and PAA (Table S2) (PDF)
