# Supplementary material for: Tunable Carrier Type of a Semiconducting 2D Metal–Organic Framework Cu3(HHTP)2
Source: ACS Appl Mater Interfaces. 2022 Mar 1;14(10):12404–11. doi: 10.1021/acsami.2c00089 (PMC9096791; doi:10.1021/acsami.2c00089)
Supplement: Supplementary file 1 — am2c00089_si_001.pdf [file am2c00089_si_001.pdf]

Supporting Information

Tunable carrier type of a semiconducting 2D Metal-Organic Framework $Cu_3(HHTP)_2$

Maria de Lourdes Gonzalez-Juarez[a], Carlos Morales[b], Jan Ingo Flege[b], Eduardo Flores[c-d], Marisol Martin-Gonzalez[c], Iris Nandhakumar[a], Darren Bradshaw*[a]*

[a]School of Chemistry, University of Southampton, Southampton SO17 1BJ, UK

Email: D. Bradshaw@soton.ac.uk; I.Nandhakumar@soton.ac.uk

[b]Applied Physics and Semiconductor Spectroscopy, Brandenburg University of Technology Cottbus–Senftenberg, Konrad-Zuse-Strasse 1, D-03046 Cottbus, Germany

[c]Instituto de Micro y Nanotecnología (CNM-CSIC), C/ Isaac Newton 8, PTM, E-28760 Tres Cantos, Spain

[d]Centro de Nanociencias y Nanotecnología (CNyN), Universidad Nacional Autónoma de México (UNAM), Ensenada, Baja California C.P. 22860, Mexico

Experimental methods

Materials

Copper(II) nitrate trihydrate, copper(II) sulfate pentahydrate (ACS reagent, ≥98.0%), potassium chloride (ACS reagent, 99.0 %), poly(methyl methacrylate) (MW ~120,000), polyacrylic acid (MW 1800), chlorobenzene (99.8%), ethanol absolute, and ammonium hydroxide solution (ACS reagent, 28.0-30.0% NH_3 basis) were purchased from Sigma-Aldrich. 2,3,6,7,10,11-hexahydroxytriphenylene hydrate (HHTP, ≥95%) was purchased from TCI Europe. Tributylmethylammonium methyl sulfate (MTBS) was purchased from Santa Cruz. All reagents and solvents were used as received without further purification. Ultrapure Milli-Q water with a resistivity higher than 18 MΩ·cm was used to prepare the solutions. Au coated silicon wafers were used as working electrodes.

Electrochemical deposition of $Cu_3(HHTP)_2$ onto Au/SiO_2

Au/SiO_2 substrates (10 x 20 mm) were used as working electrodes. Pt gauze and standard calomel electrode were used as counter and reference electrodes, respectively.

The electrolyte for the copper layer electrodeposition was made of a solution containing 0.01 M $CuSO_4$ and 0.1 KCl as supporting electrolyte. The solution was degassed with argon for 10 minutes. A potential

of -0.270 V was applied for 60 min. The anodic dissolution of Cu@Au/SiO_2 was conducted in a solution containing 2.5 mM of the ligand HHTP and 0.02 M MTBS as supporting electrolyte in a solvent ratio 80:20 EtOH:H_2O. A pontential of 0.435 V was applied for 2h.

$Cu_3(HHTP)_2$ thin films transferred with PMMA

A suspension of poly(methyl methacrylate) (PMMA) was prepared by adding 3.71 g of PMMA in 10 mL of chlorobenzene. The PMMA suspension was drop casted onto the electrodeposited $Cu_3(HHTP)_2$ film. Then, PMMA/$Cu_3(HHTP)_2$/Au/SiO_2 films were placed separately on a hot plate at 40°C and 70°C overnight. The PMMA/$Cu_3(HHTP)_2$ was carefully peeled off from the Au/SiO_2 substrate and characterised afterwards.

Characterisation methods

Grazing incidence X-ray diffraction (GIXRD) was conducted using a Rigaku Smartlab, CuKα radiation, λ=1.5406 Å. Electrical measurements were conducted using a commercial ECOPIA Hall effect measurement system. Thermovoltage measurements were conducted using a home-built Seebeck. Seebeck coefficient and electrical conductivity data is reported from the average of five samples. Morphological and thickness characterisations were carried out using a scanning electron microscope JEOL JSM-6500F. Ex-situ XPS measurements were performed on $Cu_3(HHTP)_2$ transferred films with an Omnicron EA 125 hemispherical electron analyser. Transferred MOF films were supported on Si substrates (with native oxide) using ultra-high-vacuum (UHV) compatible carbon tape. No heating and/or Ar+ sputtering has been used to avoid any change on the oxidation state or stoichiometry (by oxygen preferential sputtering). The pass energy was set to 20 eV, giving an overall resolution of about 1.2 eV. The charge effects were corrected by adjusting the Cu(II) line of the Cu 2p spectra fit at 934.7 eV. The spectra have been fitted using the XPSPeak software, version 4.1, and a Shirley background removal.

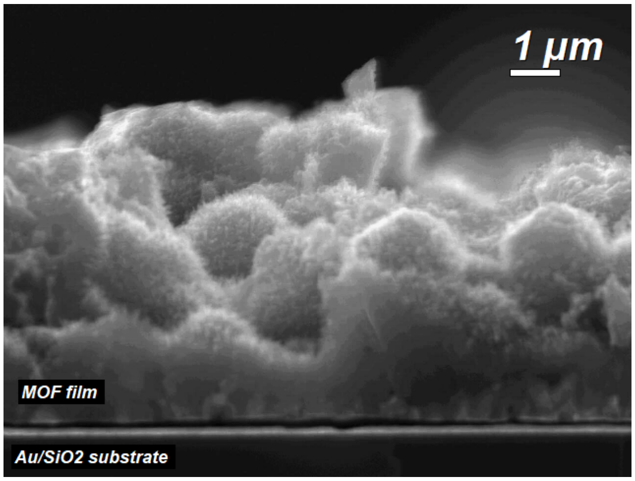

Figure S1. Cross sectional view of electrodeposited $Cu_3(HHTP)_2$ onto Au coated SiO_2

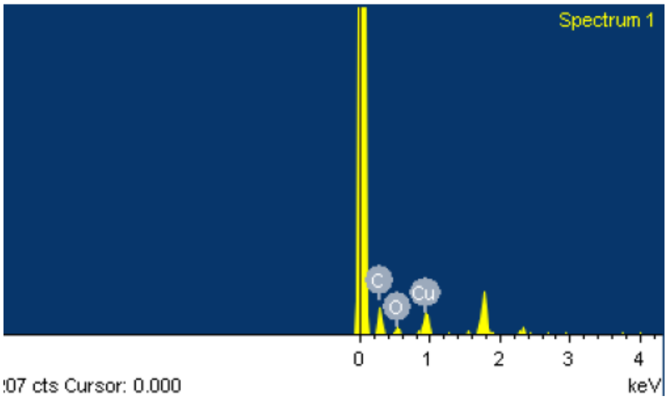

Figure S2. EDS spectrum of the electrodeposited $Cu_3(HHTP)_2$ film. The signal around 2 keV correspond to the contribution from the Au/SiO_2 substrate

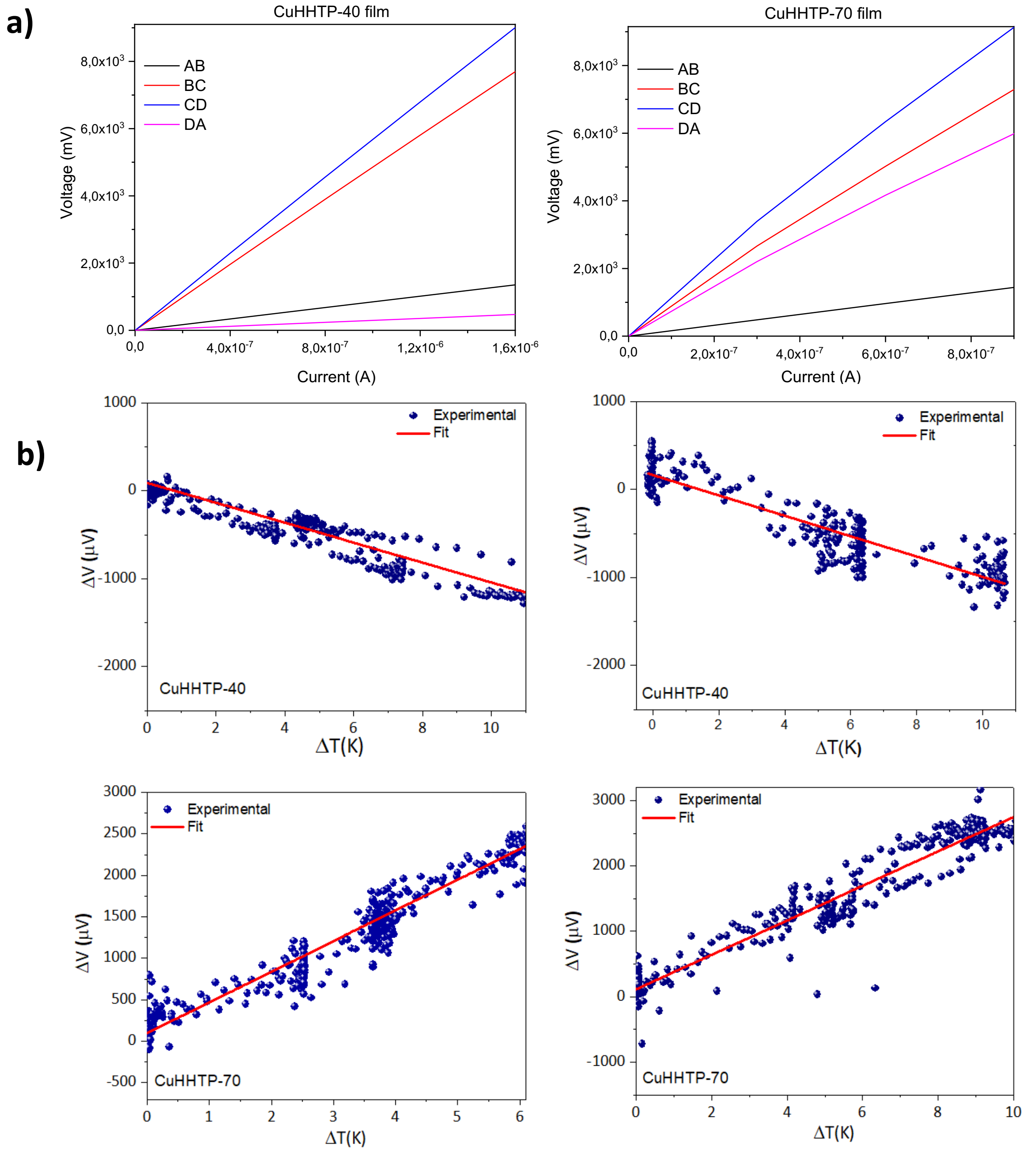

Figure S3. (a) I-V curves of transferred $Cu_3(HHTP)_2$ thin films. A, B, C and D are assigned to each of the four electrical probes placed on the sample. (b) thermovoltage measurements of transferred CuHHTP-40 and CuHHTP-70 films.

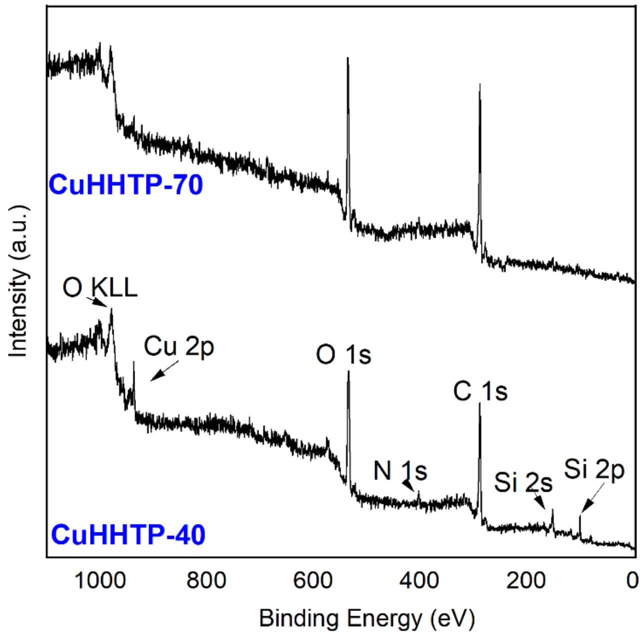

Figure S4. Survey XPS spectra of transferred CuHHTP-40 and Cu-HHTP-70 films

Sample	Cu (%)	C (%)	O (%)	N (%)
Theoretical $Cu_3(HHTP)_2$	5.9	70.6	23.5	/
CuHHTP-40	2.9	72.9	21.6	2.6
CuHHTP-70	0.7	74.9	21.5	2.9

Table S1. Atomic concentrations calculated from fitting the corresponding elemental region (Cu, C, O) in the XPS survey spectra. The presence of N is due to the supporting electrolyte used to electrodeposit the MOF films. of Cu due to the PMMA residues on the

$Cu_3(HHTP)_2$ thin films transferred with PAA

The effect of carrier type switch of $Cu_3(HHTP)_2$ thin films was also investigated by varying the polymer used as transfer agent and the solvent. For this, 700 mg of polyacrylic acid powder (MW 1800 g/mol) was dissolved in 0.3 mL of methanol. The solution was left to stir at room temperature overnight to ensure a homogenous mixture. The methodology to transfer the electrodeposited $Cu_3(HHTP)_2$ films was the same as described for PMMA. Briefly, PAA/MeOH was drop casted onto electrodeposited $Cu_3(HHTP)_2$ films and dried separately at 40°C and 70°C. These films are denoted as CuHHTP-PAA-40 and CuHHTP-PAA-70, respectively. Charge transport properties of $Cu_3(HHTP)_2$ films supported onto PAA were characterised by Seebeck and electrical measurements.

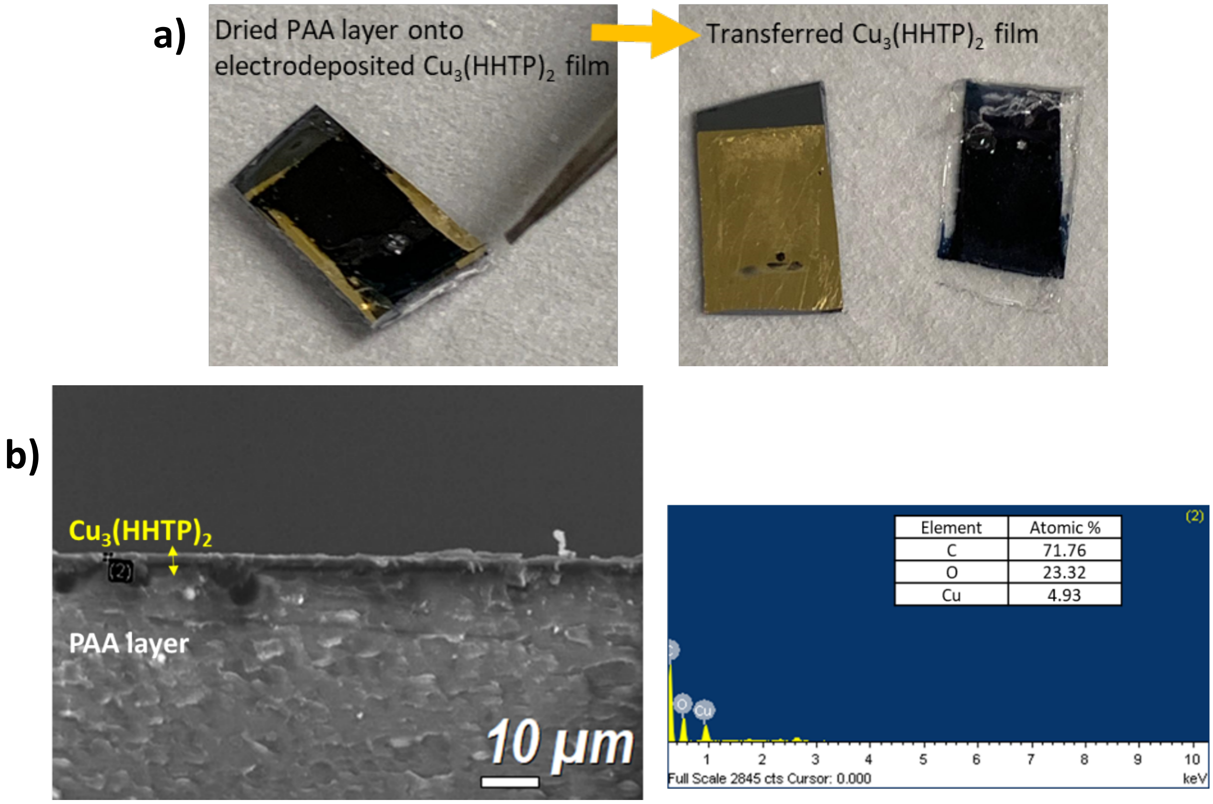

Figure S5. Photographs of $Cu_3(HHTP)_2$ film using polyacrylic acid as a transfer agent (a), and SEM cross sectional view and EDS analysis of transferred MOF film (b).

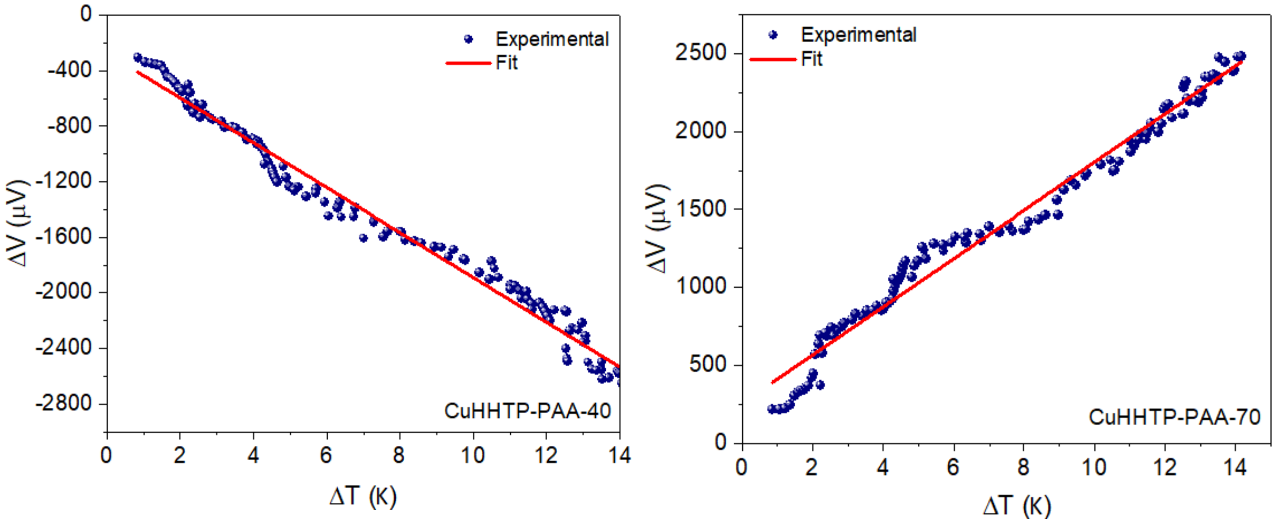

Figure S6. Thermopower data of electrodeposited $Cu_3(HHTP)_2$ films transferred using polyacrylic acid. Films were dried prior being transferred at 40°C and 70°C, respectively.

Sample	Seebeck coefficient (µV/K)	Electrical conductivity (S/cm)	Carrier concentration (cm^3)
**CuHHTP-PAA-40**	-182.06 ± 19.88	5.59×10^{-3} ± 0.00003	-9.54×10^{15}
**CuHHTP-PAA-70**	+187.72 ± 12.73	2.9×10^{-3} ± 0.0045	$+2.079 \times 10^{15}$
**CuHHTP-40**	-117 ± 13.44	2.28×10^{-3} ± 0.0007	-6.41×10^{16}
**CuHHTP-70**	+269.5 ± 21.56	4.86×10^{-4} ± 0.0012	$+2.47 \times 10^{14}$

*CuHHTP-40 and CuHHTP-70 samples were transferred using PMMA.

Table S2. Comparative charge transport data for electrodeposited $Cu_3(HHTP)_2$ films dried at 40°C and 70°C prior film transfer using PMMA and PAA.
